# Serum Interleukin (IL)-23 and IL-17 Profile in Inflammatory Bowel Disease (IBD) Patients Could Differentiate between Severe and Non-Severe Disease

**DOI:** 10.3390/jpm11111130

**Published:** 2021-11-02

**Authors:** Laura A. Lucaciu, Maria Ilieș, Ștefan C. Vesa, Radu Seicean, Shahida Din, Cristina Adela Iuga, Andrada Seicean

**Affiliations:** 1 Department of Gastroenterology and Hepatology, “Iuliu Haţieganu” University of Medicine and Pharmacy, Victor Babeș Street No. 8, 400000 Cluj-Napoca, Romania; lauraa.lucaciu@gmail.com (L.A.L.); andradaseicean@gmail.com (A.S.); 2 Department of Proteomics and Metabolomics, MedFuture-Research Centre for Advanced Medicine, “Iuliu Haţieganu” University of Medicine and Pharmacy, Victor Babeș Street No. 8, 400000 Cluj-Napoca, Romania; ilies.maria@umfcluj.ro (M.I.); iugac@umfcluj.ro (C.A.I.); 3 Department of Pharmacology, Toxicology and Clinical Pharmacology, “Iuliu Haţieganu” University of Medicine and Pharmacy, Victor Babeș Street No. 8, 400000 Cluj-Napoca, Romania; 4 Department of General Surgery, First Surgical Clinic, “Iuliu Haţieganu” University of Medicine and Pharmacy, Victor Babeș Street No. 8, 400000 Cluj-Napoca, Romania; rseicean@yahoo.com; 5 Edinburgh IBD Unit, Western General Hospital, Edinburgh EH4 2XU, UK; sdin@exseed.ed.ac.uk; 6 Department of Pharmaceutical Analysis, Faculty of Pharmacy, “Iuliu Haţieganu” University of Medicine and Pharmacy, Victor Babeș Street No. 8, 400000 Cluj-Napoca, Romania; 7 “Prof. Dr. Octavian Fodor” Regional Institute of Gastroenterology and Hepatology, Croitorilor Street No. 19-21, 400162 Cluj-Napoca, Romania

**Keywords:** Crohn’s disease, ulcerative colitis, IBD severity, interleukin-17, interleukin-23, biomarker

## Abstract

Interleukin (IL)-17 and IL-23 are crucial for mediating gut mucosal inflammation in inflammatory bowel disease (IBD), which has led to new therapeutic strategies. We assessed the relevancy of IL-17 and IL-23 serum levels as potential biomarkers towards severe IBD discrimination and disease-related complications. Sixty-two patients diagnosed with Crohn’s disease (CD) and ulcerative colitis (UC) were included. Serum IL-17 and IL-23 were measured by sandwich enzyme-linked immunosorbent assays (ELISA). IL-23 and fecal calprotectin (FCal) were significantly higher in severe CD (*p* < 0.001) and UC (*p* < 0.001 and *p* = 0.001, respectively), compared to mild or moderate. Elevated C-reactive protein (CRP) was correlated with severe disease only in CD (*p* = 0.008), whereas for UC, disease severity was associated with increased IL-17 values (*p* < 0.001). Diagnostic role of IL-23 was superior to FCal in discriminating between severe and mild to moderate CD (*p* < 0.001). IL-23 levels were also significantly higher in CD patients with intestinal complications (*p* = 0.04). Both IL-17 and IL-23 correlate with IBD severity, and IL-23 might be a promising novel biomarker for severe CD. Identifying the dominant IL pathway involved in IBD severity could serve as guidance for clinical decision-making on biologic therapy.

## 1. Introduction

Ulcerative colitis (UC) and Crohn’s disease (CD) are chronic disorders of the gastrointestinal tract with a complex etiology that involves immune, genetic, and environmental factors. Disease progression and severity are highly variable across individuals with inflammatory bowel disease (IBD), which suggests that distinct cytokine pathways may be responsible for the heterogeneity of clinical outcomes [1].

Interleukin (IL)-23/T helper (Th)-17 cytokine pathway was found to have a key role in driving gut inflammation and the development of several other chronic inflammatory diseases, such as psoriasis, rheumatoid arthritis, and multiple sclerosis [2]. Genome-wide association studies have linked IL-23 to IBD susceptibility via polymorphisms in the IL-23 receptor (IL-23-R) gene that enhances the activity of the IL-17/IL-23 pathway [3].

IL-23 levels were found to be positively correlated with other inflammatory biomarkers such as C-reactive protein (CRP) and rheumatoid factor in rheumatoid arthritis (RA) patients [4,5]. The association between disease activity score (DAS) and rheumatoid arthritis severity scale (RASS) suggested that IL-23 could be used as a biomarker to reflect RA activity [4]. Similarly, in ulcerative colitis patients, IL-23 serum levels were associated with disease severity and duration, leading to it being a possible disease diagnostic marker [6].

Furthermore, new therapeutic agents blocking components of this pathway have been developed; currently only ustekinumab is approved for the treatment of both CD and UC, but other IL-23p19 antagonists are in phase II or III development programs [7]. In contrast, IL-17A inhibition via secukinumab has been shown to worsen the outcomes in Crohn’s disease, despite its success in psoriasis [8].

This has driven further research efforts into assessing whether molecular-based stratification with biomarkers according to underlying disease mechanisms could reveal which subgroup of patients would benefit from which therapies. This is particularly important for those with an unfavorable clinical course or complex disease when treatment should be tailored accordingly. Clinical trials aim to incorporate biomarker panels into their design, which are subsequently used in post-hoc analysis, to investigate their relationship with drug response [9]. Different cytokine profiles have been studied as potential biomarkers for response to therapy [10], but their role in predicting disease course is yet to be addressed.

In this study we postulated that quantifying the IL-17 and IL-23 serum levels in patients with IBD could differentiate subgroups of patients with severe and non-severe disease, hence providing a useful clinical tool for patients’ stratification. We have tested this premise against biomarkers frequently used in clinical practice to assess disease activity, such as C-reactive protein (CRP), erythrocyte sedimentation rate (ESR), albumin, and fecal calprotectin (FCal).

## 2. Materials and Methods

### 2.1. Patients and Study Protocol

This was a pilot case–control prospective study conducted between October 2016 and December 2017. Sixty-two consecutive patients who had CD or UC, were either hospitalized for active disease or attended the day bed unit at the Regional Institute of Gastroenterology and Hepatology Cluj-Napoca, Romania, were recruited.

Eligible participants had a confirmed IBD diagnosis, based on Lennard–Jones criteria [11], serum and fecal samples collected at admission, underwent a colonoscopy or imaging study (CT scan/MRI) in the 6 months prior to admission, and agreed to participate in this study. Baseline demographic data, disease characteristics, phenotype, and the type and duration of IBD treatment (aminosalicylates, corticosteroids, immunosuppressive, and biologic agents) were recorded. Disease location, extension, and behavior were categorized according to the Montreal classification for both CD and UC. Disease activity scores were calculated using Crohn’s Disease Activity Index (CDAI) and the Mayo score, respectively [12]. The endoscopic activity was assessed by calculating the Simplified Endoscopic Activity Score for Crohn’s Disease (SES-CD) and Mayo endoscopic subscore [12].

Blood samples for inflammatory biomarkers (CRP, ESR, albumin), and fecal samples for FCal measurement were collected during admission as part of hospital protocol. Serum samples for cytokine analysis were aliquoted and stored at −80 °C.

Cut-offs for severity thresholds for mild, moderate, or severe disease were established by international consensus, according to European Crohn’s and Colitis Organization (ECCO) and American College of Gastroenterology (ACG) guidelines [12]. We classified severe disease patients as having stricturing and/or fistulizing CD, extensive UC, intestinal complications (abscess, fistula, strictures), previous IBD-related surgery, frequent relapses and need for corticosteroids, and >3 courses since diagnosis (Table S1).

The control group consisted of 15 age- and sex-matched subjects referred to our center for outpatient colonoscopy. They were selected from outpatients who did not have a history of autoimmune diseases, had a macroscopically normal colon, and negative fecal and serum inflammatory biomarkers (FCal, CRP, ESR), where available.

Patients that had a suspected or confirmed diagnosis of indeterminate colitis, infectious colitis or malignancy, patients that were pregnant at admission, or those who expressed their refusal to participate were excluded from the study.

### 2.2. Serum Cytokines Analysis

Serum IL-17 and IL-23 levels were determined using sandwich enzyme-linked immunosorbent assays (ELISA) (human IL-17 Quantikine ELISA kit D1700, human IL-23 Quantikine ELISA kit D2300B, R&D Systems, Minneapolis, MN, USA). Individual serum samples were prepared following manufacturer’s instructions and using duplicate measurements. CLARIOstar microplate reader (BMG Labtech, Ortenberg, Germany) was used for reading absorbance. Raw data were acquired using Mars Data Analysis Software (BMG Labtech, Ortenberg, Germany). For quantification (pg/mL), a 4-Parameter fit based calibration curve was generated using known concentrations of the protein standard following the protocol. Final concentrations were calculated as the mean of the two measurements. Other biomarkers (CRP, ESR, albumin and FCal) were measured at the clinic following standard procedures.

### 2.3. Statistical Analysis

Statistical analysis was performed using MedCalc Statistical Software version 19.0.7 (MedCalc Software bvba, Ostend, Belgium; https://www.medcalc.org; 2019). The mean difference between groups for IL-23 was 1433 pg/mL. For a type 1 (alpha) error of 0.05 and a type 2 (beta) error of 0.01 we calculated a sample size of 15 patients per group. Continuous data were tested for normality of distribution using the Shapiro–Wilk test and characterized by mean and standard deviation, or the median and the 25th and the 75th percentiles. Qualitative data were expressed as absolute and relative frequency. Differences between groups were verified with the Mann–Whitney or the chi-square test, as appropriate. Areas under the curve (AUC) were calculated for discriminating between mild/moderate and severe disease. A *p* value of < 0.05 was considered statistically significant.

### 2.4. Ethical Considerations

This study was performed in accordance with the WMA Declaration of Helsinki, and written informed consent was obtained from each patient before being included in the study. This study was approved by the Ethical Commission of the “Prof. Dr. Octavian Fodor” Regional Institute of Gastroenterology and Hepatology, Cluj-Napoca, Romania (decision number 16265).

## 3. Results

### 3.1. Characteristics of the Study Participants

Table 1 summarizes the demographic and disease-related characteristics of subsets of patients with severe and mild or moderate CD and UC, respectively. Based on the criteria described in the method section (Table S1), 16 patients with CD and 14 UC patients were classified as having mild-to-moderate disease, whereas 15 CD and 17 UC patients were included in the severe disease group. Sixteen patients had anti-TNF treatment, adalimumab or infliximab (Table 1). Eight patients (5 with CD and 3 with UC) had been in remission by the time of inclusion in this study; therefore, they fell in the mild or moderate disease severity category. Younger patients had severe disease in both the CD (*p* = 0.01) and UC (*p* = 0.02) groups. Intestinal complications were more frequent in patients with severe CD (*p* = 0.01). The other variables were not significantly associated with the severity of CD or UC. There were no age or gender-related statistically significant differences between healthy participants and CD or UC patients.

### 3.2. Standard Inflammatory Biomarker Levels in IBD Patients by Disease Severity vs. Healthy Participants

**Figure 1:**
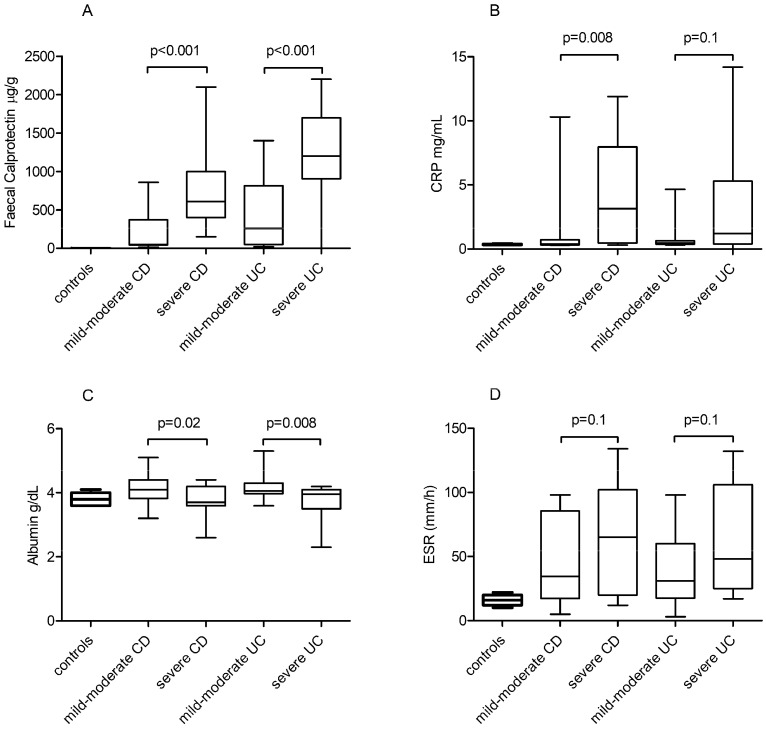
Standard inflammatory biomarker distributions in IBD patients according to disease severity: (**A**) FCal levels in mild-moderate vs. severe CD and mild-moderate vs. severe UC groups. (**B**) CRP serum levels in mild-moderate vs. severe CD and mild-moderate vs. severe UC groups. (**C**) Albumin serum levels in mild-moderate vs. severe CD and mild-moderate vs. severe UC groups. (**D**) ESR serum levels in mild-moderate vs. severe CD and mild-moderate vs. severe UC groups. Biomarker levels in controls are also shown.

Comparisons among groups between standard inflammatory biomarkers levels are shown in Figure 1 and Supplementary Table S2. FCal levels were significantly elevated in patients with severe CD and UC (*p* < 0.001) vs. mild or moderate disease groups and controls, with higher median values in UC patients. Higher CRP levels were associated with severe disease only in CD patients (*p* = 0.008). Lower albumin levels were more frequently observed in patients with severe UC (*p* = 0.008). ESR levels comparison between different groups of IBD severity did not yield significant results (*p* = 0.1).

### 3.3. IL-17 and IL-23 Quantitative Serum Levels in IBD Patients vs. Healthy Controls

IL-17 levels were significantly higher in CD patients than in controls, with a median level of 949 (IQR 400.8–2242.9) pg/mL vs. 484.8 (IQR 139–560.5) pg/mL, *p* = 0.002. IL-23 levels were significantly higher in CD patients than in controls, with a median level of 937.4 (IQR 773.3–1477.4) pg/mL vs. 371.5 (IQR 362.8–380.1) pg/mL, *p* < 0.001.

**Figure 2:**
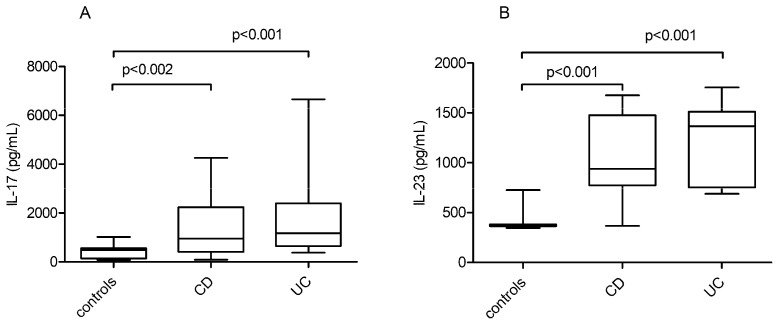
IL-17 and IL-23 quantitative serum level distribution in IBD patients and the control group: (**A**) IL-17 serum levels in UC patients, CD patients, and the control group. (**B**) IL-23 serum levels in UC patients, CD patients, and the control group.

In UC patients, IL-17 levels were significantly higher than in the control group, with a median level of 1167 (IQR 642.6–2405.7) pg/mL vs. 484.8 (IQR 139–560.5) pg/mL, *p* < 0.001. IL-23 levels were significantly higher in UC patients than in controls: 1365.1 (751.7–1512) pg/mL vs. 371.5 (362.8; 380.1) pg/mL, *p* < 0.001 (Figure 2 and Table S2).

### 3.4. IL-17 and IL-23 Quantitative Serum Levels Associated with IBD Severity vs. Healthy Controls

**Figure 3:**
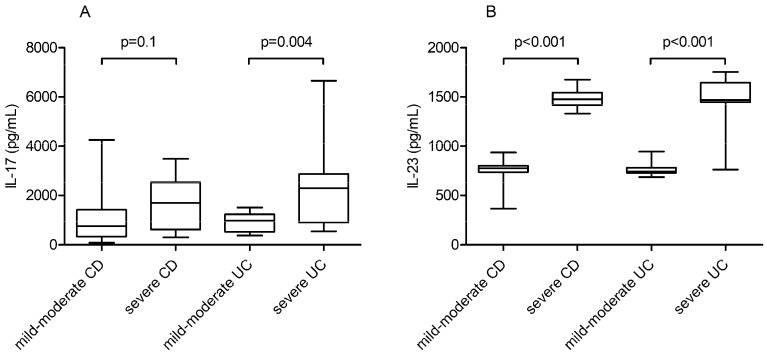
IL-17 and IL-23 quantitative serum level distribution in IBD patients according to disease severity: (**A**) IL-17 serum levels in mild-moderate vs. severe CD groups and mild-moderate vs. severe UC groups. (**B**) IL-23 serum levels in mild-moderate vs. severe CD groups and mild-moderate vs. severe UC groups.

IL-23 serum levels were significantly elevated in patients with severe CD and UC (*p* < 0.001), whereas IL-17 levels were significantly higher only in UC patients with severe disease (*p* < 0.001). In patients with severe CD, serum IL-17 levels were not significantly higher than in those with mild or moderate disease (*p* = 0.1) (Figure 3 and Table S2).

### 3.5. Discriminating between Severe and Mild or Moderate IBD according to Biomarker Cut-Off Value

We calculated the performance of cut-off values for the biomarkers that discriminated between mild/moderate and severe disease (Table 2) using ROC (Receiver Operating Characteristic) Curve. Cut-off values for each biomarker were established where sensitivity and specificity were maximal. A significantly higher area under the ROC curve (AUC) of IL-23 for discriminating patients with severe CD was found, as compared to the AUC of CRP, FCal, or albumin (*p* = 0.003; *p* = 0.04; *p* = 0.01, respectively) (Table 2). For the identification of severe UC cases, the IL-23′s AUC was only slightly better than FCal (*p* = 0.09), but significantly higher than the AUC of IL-17 or albumin (*p* = 0.02; *p* = 0.01, respectively). The specificity of IL-23 in discriminating severe UC cases was found to be higher compared to the FCal. The assessment of IL-17 and IL-23 levels according to biologic therapy has not yielded significant results (*p* = 0.274 and *p* = 0.184, respectively).

### 3.6. Assessment of CD Disease Complications according to Standard Inflammatory Biomarkers vs. IL-17 and IL-23 Serum Levels

We compared the median biomarker levels in CD patients with and without intestinal complications such as enteral fistulae, abscesses, or need for surgery (Table 3). IL-23 and, to a lesser degree, IL-17 had significantly higher levels in patients with intestinal complications (*p* = 0.04 and *p* = 0.05, respectively). There was a positive point-biserial (Pearson’s) correlation between both IL-17 and IL-23 and the presence of intestinal complications (rpb = 0.085 and 0.243); this was statistically significant for IL-23 (*p* = 0.04) but not for IL-17 (*p* = 0.51).

## 4. Discussion

In order to achieve the goal of precision medicine, a clinical tool towards IBD patient stratification is urgently needed. So far, a molecular-based biomarker that could differentiate between subgroups of patients with severe and non-severe IBD is absent. We have therefore shown that serum levels of IL-17 and IL-23 could become a valuable biomarker for assessing disease severity subtypes in both CD and UC, by testing these molecules against biomarkers frequently used in clinical practice such as CRP, ESR, albumin, and FCal.

The results of our study highlight that serum IL-23 levels showed superior diagnostic potential to that of dedicated inflammatory biomarkers for IBD, such as FCal, CRP, and albumin. In contrast, higher IL-17 serum levels only demonstrated a modest diagnostic performance for both severe UC and CD. In addition, IL-17 and IL-23 serum levels were significantly more elevated in IBD patients compared to the control group.

It is known that cytokines amplify and sustain the inflammatory response in IBD; hence, elevated concentration in biological fluids is expected. This also indicates the pathway activation that drives inflammation and may lead to disease progression [13]. IL-23 is released by monocytes/macrophages/dendritic cells in response to bacterial stimulation, and subsequent interaction with its heterodimeric IL-23 receptor (IL-23R) leads to production of inflammatory mediators (IL-17, IL-22, granulocyte-macrophage colony-stimulating factor (GM-CSF), TNF-α) through JAK-STAT pathway activation [14,15]. IL-17 acts as a proinflammatory cytokine produced by Th-17 cells [16] that has divergent functions, contributing to chronic inflammation via recruitment and activation of inflammatory mediators, and host defense against extracellular bacterial and fungal infections [17].

Elevated levels of IL-17 and IL-23 in IBD patients have previously been reported separately [6,18,19,20,21]. Fujino et al. [18] were the first to demonstrate a positive correlation between elevated levels of IL-17 in the inflamed mucosa and active lesions of IBD patients, as well as elevated serum levels of IL-17 and disease activity in both CD and UC. Similarly, Jiang et al. reported increased expression of Th-17 cytokines (IL-17, IL-21, and IL-22) in the intestinal mucosa in active IBD patients [19].

Studies assessing IL-23 expression in human IBD have also shown increased IL-23 production by lamina propria macrophages from CD patients but not in UC [20]. These findings are supported by studies in patients with concomitant inflammatory conditions, such as arthritis and sacroileitis [21], which reported higher IL-23 levels in CD rather than in those with UC. In contrast, Mirsattari et al. [6] showed that higher IL-23 levels corelate with both duration and severity of the disease (based on a modified Mayo score) but only in UC patients. These findings confirm the important role of IL-23 in IBD pathogenesis, making it not only an important therapeutic target but also a potential biomarker for IBD severity and prognosis. 

Understanding and addressing disease prognosis in IBD is the basis for developing a precision medicine approach [22]. Stratifying patients in a timely manner and individualizing therapy is a continuous challenge in clinical practice. Many clinical parameters, such as serological markers, disease location, disease behavior, age and lifestyle, have been found to be associated with disease severity [23]. However, most of these predictors derive from observational studies and are not specific enough to alter early disease management. Therefore, the need for reliable biomarkers is paramount.

We obtained a high AUC for IL-23 in detecting CD patients prone to more severe disease. Furthermore, this was superior to the standard inflammation markers used in the clinical practice (CRP, FCal, albumin), therefore acting as potential noninvasive biomarker for identifying a complex disease phenotype in patients with CD.

While previous studies assessing the expression of IL-17 and IL-23 in IBD patients have classified patients according to “disease severity”, they have primarily relied on disease activity scores that have been symptom based at a point in time; however, patients may have severe disease, warranting aggressive therapies even if their disease presentation is not severe. For example, patients with extensive steroid-dependent ulcerative colitis or Crohn’s disease refractory to immunosuppressive therapies, with mild symptoms but on high doses of corticosteroids, can be considered [24]. We have therefore taken into consideration a more comprehensive approach when defining disease severity, beyond merely clinical presentation, such as disease burden and structural damage.

In CD patients IL-23 and IL-17 were significantly higher in patients who developed intestinal complications, such as fistulae, abscesses, and need for surgery at a certain point in time. Moreover, the point-biserial correlation coefficient showed a statistically significant correlation between IL-23 and CD patients with intestinal complications. It was not possible to comparatively assess complications for UC patients due to the small number of patients that had disease-related complications. Several biomarkers that are well established in clinical practice have been used to predict a complicated disease course in IBD. FCal has a crucial role in IBD diagnosis and disease activity assessment, prediction of relapse, response to therapy [25], disease extension, and severity [26]. Our findings have demonstrated inferior diagnostic value to that of IL-23 in discriminating a more severe CD outcome; in the UC cohort, however, the sensitivities for IL-23 and FCal were comparable, but with higher specificity for IL-23.

CRP is one of the most reliable and widely used, in conjunction with FCal, to assess IBD flares in clinical practice. Previous studies have shown that a high CRP is indicative of severe disease complications (perforations, abscesses, and fistula) in CD [25,26], whereas in UC it is correlated with disease activity and severe symptoms but not with histologic inflammation [27]. In our study, CRP was inferior to IL-23 in accurately assessing disease severity in both CD and UC groups.

The ESR is a non-specific measure of systemic inflammation, and it can be used to monitor the acute-phase response of disease after 24 h [28]. We have shown that elevated ESR levels had no diagnostic value for a more severe disease course and was not correlated with the occurrence of intestinal complications in CD patients.

Serum albumin had the lowest specificity for diagnosing severe disease when compared to IL-17 and IL-23, amongst the other inflammatory biomarkers. Previous studies have shown that hypoalbuminemia in CD patients could predict poor postoperative outcomes [29], whereas in those with acute severe UC it is correlated with treatment failure, higher colectomy rate [30], and non-response to anti-TNF therapy [31]. In our CD patients, however, albumin levels had no significant association with disease-related complications.

The predictive potential of quantifying serum cytokines in IBD patients has previously been explored in the development of primary non-response in patients treated with anti-TNFα [32] and vedolizumab [33]. Furthermore, a transcriptomic analysis of the colonic mucosa in patients refractory to anti-TNF therapy showed that IL-23 gene expression is regulated upon anti-TNF blockade [34]; therefore, anti-TNF therapy is already effective in suppressing IL-23. It has been postulated that non-responders to anti-TNF might have less to gain from further IL-23 targeting. This is supported by the landmark UNITI-1, UNITI-2, and UNITI-IM studies that have shown better response rates in TNF-naive than in TNF experienced populations (54–58% vs 34%, respectively) [35]. This suggests that stratifying patients with different degrees of IBD severity, based on IL-17 and IL-23 serum profiling, could eventually be useful in guiding the choice of biologic therapy. This would entail, however, longitudinal comparisons between groups undergoing different targeted therapies.

This is the first study to explore the use of IL-17 and IL-23 in stratifying IBD patients by disease severity, in comparison with standard inflammatory tests used in clinical practice. Our results highlight that IL-23 was particularly efficient in differentiating IBD patients with a severe disease phenotype and that its performance surpassed that of faecal calprotectin. By also demonstrating a significant association with CD intestinal complications, we believe that IL-23 could become a reliable tool for stratifying CD patients since diagnosis. In comparison with the data already published, our findings have taken a step forward towards implementing these potential biomarkers in clinical practice.

So far, our results are based only on a small number of patient samples and a single time-point analysis. Serum cytokines and standard biomarker serum levels could not be included for comparison to the histological findings in the study as they were not assessed at the same time point. However, long term follow-up data for the IBD and UC patients are the focus of our future research, and the current findings will be applied with respect to disease progression in a future study.

## 5. Conclusions

Serum IL-23 levels were higher in both CD and UC patients and more effective than FCal in identifying the group with the highest disease severity. A significant correlation with intestinal complications was demonstrated in CD patients; therefore, IL-23 could become a useful biomarker in precision medicine. IL-17 was more elevated in UC patients with severe disease rather than CD but yielded lower diagnostic accuracy for disease severity when compared to other biomarkers. Further larger studies are necessary to establish whether the assessment of these cytokines in unresponsive patients to anti-TNF therapy could guide the choice of second-line biologics.

## Figures and Tables

**Table 1:** Baseline characteristics of the study participants.

		Mild or Moderate CD (n = 16)	Severe CD (n = 15)	*p* Value	Mild or Moderate UC (n = 14)	Severe UC (n = 17)	*p* Value	Controls (n = 15)
Age (Years), Mean ± SD		40.62 ± 10.54	31.8 ± 9.01	0.01	52.14 ± 18.79	37.47 ± 13.08	0.01	30.8 ± 6.12
Gender (F), n (%)		11 (68.75)	7 (46.67)	0.3	3 (21.43)	4 (23.53)	1	6 (5.3)
CDAI/Mayo Score, Median (IQR)		105(80.5–147.75)	368 (294–434)	<0.001	6 (2.25–7)	10 (10–12)	<0.001	
**Age at Diagnosis, n, % (Montreal Classification, CD)**								
A1 (n, %): <16		0 (0)	2 (13.33)					
A2 (n, %): 17–40		13 (81.25)	13 (86.67)	1				
A3 (n, %): >40		3 (18.75)	0 (0)					
**Location, n, % (Montreal Classification, CD)**								
L1: Ileum		10 (62.5)	5 (33.33)					
L2: Colon		2 (12.5)	2 (13.33)	0.2				
L3: Ileocolonic		4 (25)	8 (53.33)					
**Location, n, % (Montreal Classification, UC)**								
E1 (Distal Colitis)		-	-		1	1		
E2 (Left-Sided Colitis)		-	-		10	13		
E3 (Pancolitis)		-	-		3	3		
**Behaviour, n, % (Montreal Classification, CD)**								
B1: Non-Stricturing, Non-Penetrating		11 (68.75)	0 (0)					
B2: Stricturing		2 (12.5)	6 (40)	<0.001				
B3: Penetrating		2 (12.5)	7 (46.67)					
Mayo Endoscopic Score, Median, (IQR)					1 (0.5–1)	3 (2–3)		
SES-CD, Median (IQR)		5 (4–7)	13.5 (12–17.5)					
**Complications, n, %, CD and UC**								
Intestinal Complications, n (%)	With	6 (37.5)	13 (86.67)	0.01	0 (0)	2 (11.76)		
	Without	10 (62.5)	2 (13.3)		14 (100)	15 (88.2)		
Fistula, n (%)		0 (0)	1 (5.88)	0.08	-	-	-	
Stenosis, n (%)		2 (12.5)	7 (46.7)	0.05	-	1 (5.9%)	1	
Abscess, n (%)		0 (0)	2 (13.33)	0.2	-	-	-	
Extraintestinal Complications, n (%)		3 (18.75)	2 (13.33)	1	-	1 (5.9)	1	
Anti-TNF Therapy, n (%)		5 (31.25)	4 (26.67)	1	3 (21.43)	4 (25)	1	

CD, Crohn’s disease. SD, standard deviation. UC, ulcerative colitis. SD, standard deviation. IQR, interquartile range.

**Table 2:** AUC values for assessing disease severity.

Variable		AUC (CI 95%)	Cut-off	Sensitivity (CI 95%)	Specificity (CI 95%)	*p* Value
**CD**	Albumin	0.733 (0.545–0.875)	3.7 g/dL	93.75 (69.8–99.8)	53.33 (26.6–78.7)	0.010
	CRP	0.781 (0.597–0.909)	1.26 mg/L	73.33 (44.9–92.2)	87.50 (61.7–98.4)	0.001
	FCal	0.877 (0.709–0.967)	50 µg/g	100 (78.2–100)	62.5 (35.4–84.8)	<0.001
	IL23	1 (0.888–1.000)	937.4 pg/mL	100 (78.2–100.0)	100 (79.4–100.0)	<0.001
	IL-17	0.667 (0.475–0.825)	>1536.15 pg/mL	53.33 (26.6–78.7)	87.50 (61.7–98.4)	0.010
**UC**	Albumin	0.773 (0.588–0.903)	3.7 g/dL	92.86 (66.1–99.8)	47.06 (23.0–72.2)	<0.001
	FCal	0.857 (0.685–0.956)	400 µg/g	94.12 (71.3–99.9)	71.43 (41.9–91.6)	<0.001
	IL-17	0.803 (0.621–0.923)	1512.05 pg/mL	64.71 (38.3–85.8)	100 (76.8–100)	<0.001
	IL-23	0.979 (0.851–1)	946.11 pg/mL	94.12 (71.3–99.9)	100 (76.8–100)	<0.001
	CRP	0.645 (0.453–0.808)	>0.89 mg/L	52.94 (27.8–77.0)	85.71 (57.2–98.2)	0.010

AUC, Area Under the Curve. CI, Confidence Interval. CD, Crohn’s disease. UC, Ulcerative Colitis. FCal, Faecal Calprotectin.

**Table 3:** IL-17, IL-23, and standard inflammatory biomarker levels in patients with or without CD-related complications.

Biomarkers’ Serum Levels (Median, IQR)	Intestinal Complications		*p* Value
	Yes (n = 19)	No (n = 12)	
CRP	1.26 (0.36–3.80)	0.44 (0.33–6.44)	0.60
Albumin	4 (3.7; 4.2)	3.9 (3.65; 4.37)	0.90
Calprotectin	400 (150–850)	135 (50–400)	0.20
ESR	52 (21–82)	27 (13–90)	0.40
IL-17	1449.05 (708.50–2531.60)	638.77 (242.93–1322.96)	0.05
IL-23	1417.01 (799.22–1486.13)	797.06 (744.14–912.63)	0.04

IQR, Interquartile range. CRP, C-reactive protein. ESR, Erythrocyte sedimentation rate. IL, Interleukin.

## Supplementary Materials

The following are available online at https://www.mdpi.com/article/10.3390/jpm11111130/s1, Table S1: Classification of study participants according to disease severity, Table S2: Comparison between groups according to IL-17, IL-23, and standard inflammatory biomarkers levels.
