# Supplementary material for: Serum Interleukin (IL)-23 and IL-17 Profile in Inflammatory Bowel Disease (IBD) Patients Could Differentiate between Severe and Non-Severe Disease
Source: J Pers Med. 2021 Nov 2;11(11):1130. doi: 10.3390/jpm11111130 (PMC8621192; doi:10.3390/jpm11111130)
Supplement: Supplementary file 1 [file jpm-11-01130-s001.zip › Supplementary Table S1_Lucaciu et al.pdf]

Supplementary Table S1. Classification of study participants according to disease severity

Criteria/ Severity	CD		UC	
	Mild or moderate	Severe	Mild or moderate	Severe
CDAI/ Mayo score	150 - 220	220 - 450	3 - 10	11 - 12
SES-CD/ Mayo endoscopic	3 - 15	> 16	2-6	➢ 7
Disease extension	-	-	-	Extensive/ Pancolitis
Intestinal complications	-	Abscess/ Fistula/ Strictures	-	-
IBD-related surgery	-	Yes	-	Yes
Frequent relapses	-	> 3 courses of steroids	-	> 3 courses of steroids

[1]CD, Crohn's disease. UC, Ulcerative colitis. CDAI, Crohn's disease activity index. SES-CD, Simple Endoscopic Score for Crohn's disease.
