# Supplementary material for: Serum Interleukin (IL)-23 and IL-17 Profile in Inflammatory Bowel Disease (IBD) Patients Could Differentiate between Severe and Non-Severe Disease
Source: J Pers Med. 2021 Nov 2;11(11):1130. doi: 10.3390/jpm11111130 (PMC8621192; doi:10.3390/jpm11111130)
Supplement: Supplementary file 1 [file jpm-11-01130-s001.zip › Supplementary Table S2_Lucaciu et al.pdf]

Supplementary Table S2. Comparison between groups according to IL-17, IL-23 and standard inflammatory biomarkers levels

	CD			UC		
**Variables (Median, IQR)**	**Mild or moderate (n=16)**	**Severe (n = 15)**	**p value**	**Mild or moderate (n = 14)**	**Severe (n = 17)**	**p value**
**Albumin (g/dL)**	4 (3.82; 4.4)	3.7 (3.6; 4.2)	0.02	4 (3.97; 4.3)	3.9 (3.5; 4)	0.008
**ESR**	34.5 (17.25; 85.5)	65 (20; 102)	0.1	31 (17.5; 60)	48 (25; 106)	0.1
**CRP (mg/L)**	0.39 (0.32; 0.72)	3.1 (0.46; 7.9)	0.008	0.47 (0.37; 0.65)	1.2 (0.39; 5.29)	0.1
**Calprotectin (μg/g)**	50 (45; 370)	640 (400; 1000)	<0.001	260 (50; 812.5)	1200 (905; 1700)	0.001
**IL-17 (pg/mL)**	764.52 (528.43; 1222.05)	1695.00 (665.35; 2535.75)	0.1	976.47 (528.43; 1222.05)	2304.60 (1011.30; 2815.42)	0.004
**IL-23 (pg/mL)**	777.63 (744.15; 800.31)	1477.49 (1434.29; 1537.97)	<0.001	745.23 (731.19; 946.11)	1468.85 (1451.57; 1598.46)	< 0.001

[1]CD, Crohn's disease; UC, ulcerative colitis. IQR, Interquartile range. ESR, Erythrocyte sedimentation rate. CRP, C-reactive protein. IL, Interleukin.
